# Erythème flagellé secondaire à la Bléomycine: à propos de deux observations

**DOI:** 10.11604/pamj.2018.30.263.10158

**Published:** 2018-08-07

**Authors:** Asmae Lahlou, Salim Gallouj, Fatima-Zohra Mernissi

**Affiliations:** 1 University Hospital Hassan II, Dermatology Department, Fèz, Morocco

**Keywords:** Bléomycine, érythème flagellé, cytotoxicité, Bléomycin, flagellate erythema, cytotoxicity

## Abstract

La bléomycine est un antibiotique antitumoral. Elle peut être responsable d'effets secondaires variables en particulier cutanés et pulmonaires. Certains sont rares, mais spécifiques de la bléomycine. Nous rapportons l'observation de deux malades âgés respectivement de 35 et 38 ans ayant présentés une toxidermie à la bléomycine a type d'érythème flagellé après administration de bléomycine pour un choriocarcinome et un lymphome malin non hodgkinien L'érythème flagellé est spécifique de la bléomycine, il complique 10 à 30% des traitements dans un délai de quelques heures à 9 semaines. Il est indépendant de la dose, de la voie d'administration et de la nature de la lésion traitée. Ces lésions apparaissent après une dose cumulative de 90 à 285 mg. Cliniquement, il s'agit de bandes linéaires avec un aspect flagellé prédominantes sur tout le tronc et les parties proximales des membres. L'éthiopathogénie est incertaine. L'évolution est marquée par une résolution spontanée des lésions à l'arrêt du traitement parfois au prix d'une pigmentation linéaire cicatricielle, ou une sclérose résiduelle.

The bléomycine is an antitumor antibiotic. It can be responsible for in particular cutaneous and pulmonary variable side effects. Some are rare, but specific of the bléomycine. We report the observation of two patients aged respectively of 35 and 38 years old, having presented a toxidermy at the bléomycine has standard erythema whipped after administration of bléomycine for a choriocarcinomist and a malignant lymphoma not hodgkinien the whipped erythema is specific of bléomycine, it complicates 10 to 30% of the treatments within a few hours to 9 weeks. It is independent of the amount, the route of administration and the nature of the treated lesion. These lesions appear after a cumulative amount from 90 to 285 Mg. Clinically, they are linear bands with an aspect whipped prevalent on all the trunk and the proximales parts of the members. The éthiopathogénie is dubious. The evolution is marked by a spontaneous resolution of the lesions to the stop of the treatment sometimes the price of a cicatricial linear pigmentation, or a residual sclerosis.

## Introduction

La bléomycine, initialement extraite du champignon Streptomycesverticillus [1] C'est un antibiotique antitumoral [2]. Il a, à faible concentration, un effet cytostatique en inhibant les mitoses et, à forte concentration, il bloque l'incorporation de thymidine dans l'ADN. De la sorte, le médicament arrête la phase S du cycle cellulaire et il provoque le clivage de l'ADN [2, 3]. Elle est indiquée dans le traitement de cancers variés : carcinomes cutanés épidermoïdes, lymphomes malins, tumeurs germinales. Plus accessoirement, elle est utilisée comme traitement topique des cicatrices chéloïdes et des verrues plantaires [4]. Elle peut être responsable d'effets secondaires variables en particulier la toxicité cutanée et pulmonaire expliquée par l'absence dans ces deux tissus d'hydrolase, capable d'inactiver la bléomycine, entraînant ainsi une importante concentration du médicament dans ces deux organes [5, 6]. Certains effets secondaires cutanés sont plus exceptionnels, comme l'illustre ces observations.

## Patient et observation

**Figure 1:**
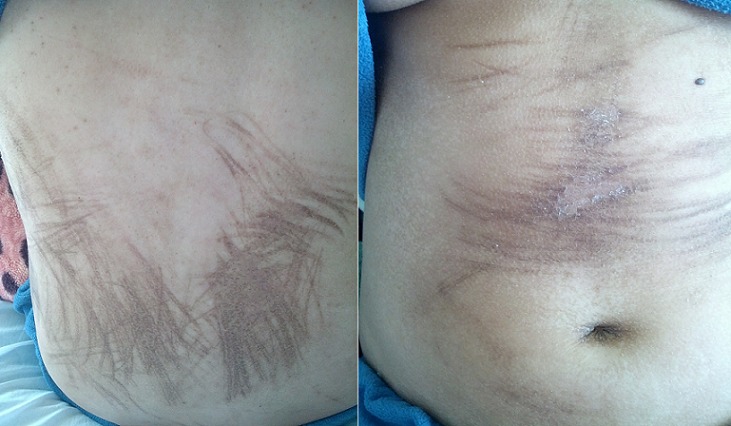
Erythème flagellé secondaire à la Bléomycine au niveau de l’abdomen

**Observation 1**: J.B âgée de 35 ans sans ATCDS pathologiques notables suivie pour choriocarcinome pour lequel elle a été mise sous polychimiothérapie a base de Bléomycine, Etoposide et cysplastine. Elle a présenté après trois cures une éruption érythémateuse non prurigineuse du dos. L'examen clinique objectivait des macules pigmentées linéaires flagellée au niveau de l'abdomen et du dos surmontées de fines squames Figure 1. Le diagnostic d'érythème flagellé secondaire à la Bléomycine a été retenu. La patiente est décédée 2 jours après, et ceci, suite à l'évolution de sa maladie.

**Observation 2**: M.C âgée de 38 ans sans ATCDS pathologiques suivie pour LMNH pour lequel elle a été mise sous polychimiothérapie à base d'Adriamycine, Bléomycine, Vinblastine, Dacarbazine. Elle a présenté après six mois de traitement un érythème flagellé du dos et de l'abdomen, avec une mélanonychie diffuse, et une pigmentation gingivale.

## Discussion

Après son administration, la bléomycine est rapidement inactivée dans la plupart des tissus par une hydrolase. Les poumons et la peau sont dépourvus de cette enzyme, ce qui explique qu'ils soient le site électif d'expression de la toxicité de cette substance [7]. Les effets secondaires cutanés sont variés. Certains, comme l'alopécie, sont communs à beaucoup de chimiothérapie. D'autres sont bien plus caractéristiques de la bléomycine. C'est le cas de l'hyperpigmentation, prédominant au niveau des zones de pression et des ongles, des plaques sclérodermiformes, des nécroses digitales compliquant un syndrome de Raynaud, de l'hidradénite neutrophilique eccrine ou encore de l'érythème flagellé [8]. Ce dernier est spécifique de la bléomycine, bien qu'il puisse également compliquer un traitement par péplomycine (un dérivé de la bléomycine) [8]. L'érythème flagellé et la pigmentation linéaire sont décrits pour la première fois par Moulin en 1970 [9]. Ces réactions sont spécifiques du traitement par la bléomycine [10, 11] et indiquent une susceptibilité individuelle, elle surviennent indépendamment de la dose, de la voie d'administration. Le délai moyen entre l'administration du traitement et le début de l'éruption varie de quelques heures à six mois [8]. Le diagnostic reste clinique. Les lésions prédominent au niveau du tronc et des parties proximales des membres [11, 12]. Nos patientes avaient présenté un érythème linéaire flagellé au niveau du dos et l'abdomen juste après la 3^ème^ cure pour la première patiente et le 6^ème^ mois de traitement pour la deuxième. ce qui est conforme aux données de la littérature. L'aspect histologique typique inclue une hyperkératose, une parakératose, un infiltrat dermique lymphohistocytaire, des mélanophages dans les capillaires dermiques et des vésiculopustules, parfois il s'agit d'une vasculite lymphocytaire [12-14]. L'étiopathogénie est incertaine, toutes les hypothèses se sont basées sur les aspects histologiques ou ultrastructuraux trouvés. Certains auteurs ont évoqué un mécanisme traumatique par le grattage suite à un prurit intense, postinflammatoire ou une vasodilatation responsable d'une concentration accrue de la bléomycine au niveau de la peau qui atteint directement les kératinocytes. D'autres auteurs, suggèrent que l'hyperpigmentation est de nature post-inflammatoire suite à une incontinence pigmentaire en rapport avec les lésions épidermiques (une accentuation localisée de la mélanogénèse a été objectivée par l'étude ultrastructurale) [2, 14]. Ces auteurs supposent que la bléomycine ralenti le cycle kératinocytaire avec un blocage relatif des mélanocytes à la phase de synthèse de pigment [2]. Enfin Lindae et Nikoloff [6] suggèrent que l'absence de l'hydrolase dans la peau entraîne une accumulation de la bléomycine qui, par un effet direct sur les kératinocytes, provoque l'infiltrat inflammatoire dermique. L'évolution est marquée par une résolution spontanée des lésions à l'arrêt du traitement parfois au prix d'une pigmentation linéaire cicatricielle, comme chez nos patientes, ou une sclérose résiduelle [15].

## Conclusion

L'érythème flagellé est considéré comme complication spécifique de la bléomycine qui peut survenir indépendamment de la dose ou du mode d'administration, cela justifie une surveillance attentive des patients recevant une chimiothérapie à base de bléomycine.

## Conflits d’intérêts

Les auteurs déclarent qu'il n'existe aucun confit d'intérêts.
